# Phages in the Gut Ecosystem

**DOI:** 10.3389/fcimb.2021.822562

**Published:** 2022-01-04

**Authors:** Michele Zuppi, Heather L. Hendrickson, Justin M. O’Sullivan, Tommi Vatanen

**Affiliations:** ^1^ The Liggins Institute, University of Auckland, Auckland, New Zealand; ^2^ The School of Natural and Computational Sciences, Massey University, Auckland, New Zealand; ^3^ The Maurice Wilkins Centre, The University of Auckland, Auckland, New Zealand; ^4^ MRC Lifecourse Epidemiology Unit, University of Southampton, Southampton, United Kingdom; ^5^ The Broad Institute of MIT and Harvard, Cambridge, MA, United States

**Keywords:** gut microbiome, bacteriophage, prophage, microbial ecology, metagenomics

## Abstract

Phages, short for bacteriophages, are viruses that specifically infect bacteria and are the most abundant biological entities on earth found in every explored environment, from the deep sea to the Sahara Desert. Phages are abundant within the human biome and are gaining increasing recognition as potential modulators of the gut ecosystem. For example, they have been connected to gastrointestinal diseases and the treatment efficacy of Fecal Microbiota Transplant. The ability of phages to modulate the human gut microbiome has been attributed to the predation of bacteria or the promotion of bacterial survival by the transfer of genes that enhance bacterial fitness upon infection. In addition, phages have been shown to interact with the human immune system with variable outcomes. Despite the increasing evidence supporting the importance of phages in the gut ecosystem, the extent of their influence on the shape of the gut ecosystem is yet to be fully understood. Here, we discuss evidence for phage modulation of the gut microbiome, postulating that phages are pivotal contributors to the gut ecosystem dynamics. We therefore propose novel research questions to further elucidate the role(s) that they have within the human ecosystem and its impact on our health and well-being.

## Introduction

The gastrointestinal tract (GIT) of humans and many other animals hosts a complex ecosystem inhabited by a plethora of different microorganisms, that include bacteria, fungi, archaea, protozoa, and viruses (Martín et al., 2014). Multiple factors affect gut microbial communities and contribute to the complexity of this ecosystem. These factors include (but are not limited to) the anatomy of the GIT, peristaltic movements, the mucus layer and its shedding, host-produced compounds (e.g. bile acids or gastric juice), the constant influx of new microorganisms and nutrients through diet, and the host immune system (Thursby and Juge, 2017; Shkoporov and Hill, 2019).

The microbial communities (microbiomes) in the gut are involved in regulating many aspects of the host’s well-being through the mediation of nutrient absorption, synthesis of vitamins and neurotransmitters, the development and modulation of the immune system, and modifying resistance against pathogens, among others (Heintz-Buschart and Wilmes, 2018). Therefore, it is unsurprising that disturbances in this ecosystem, reflected by alterations in the microbial communities that inhabit it, have been connected to multiple diseases, from gut inflammation to neurological disorders (Mulak and Bonaz, 2015; Thursby and Juge, 2017).

To date, the majority of the reported microbial impacts on the gut ecosystem and host health have been connected to the bacterial component of the microbiome (Chow et al., 2010). Recently, viruses that infect bacteria, namely bacteriophages or, phages for short, have been gaining attention as potential modulators of the gut ecosystem due to their ability to affect bacterial communities. Most peculiarly, the influence of phages on the gut ecosystem seems to extend beyond their direct impacts on bacterial populations, extending to modulation of the host immune system (Sausset et al., 2020). Furthermore, alterations in the gut phage population have been connected to gastrointestinal diseases on multiple occasions (Norman et al., 2015; Manrique et al., 2016; Draper et al., 2018; Zuo et al., 2018; Clooney et al., 2019; Park et al., 2019), highlighting their contribution to gastrointestinal health. In this review, we postulate that phages play a major role in the gut ecosystem dynamics through an intricate network of interactions with both the gut bacterial community and the host immune system.

### Phages: An Overview

**Figure 1:**
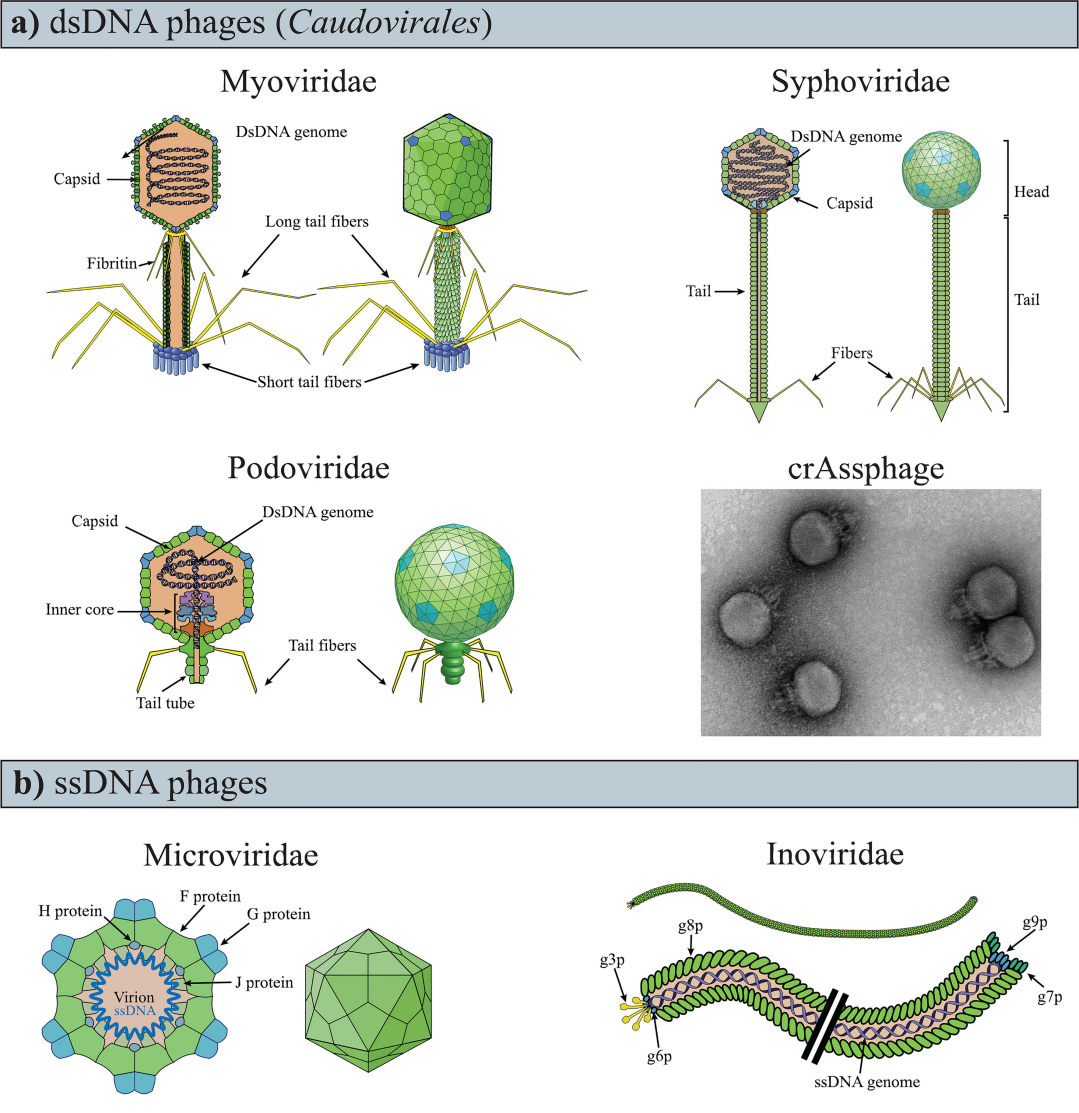
Variability of phage genomes and capsids within the known gut phages. **(A)** dsDNA phages of the *Caudovirales* order have a polyhedral capsid to which is attached a tail, the representative feature of the order. **(B)** TEM image of crAssphage (ΦcrAss001), negatively contrasted with uranyl acetate. Image modified from ref (Shkoporov et al., 2018a). **(C)** ssDNA phages are currently recognized as having either icosahedral or filamentous capsids (i.e., *Microviridae* and *Inoviridae*, respectively). The drawings of the phages were obtained and modified from ViralZone, SIB Swiss Institute of Bioinformatics (Hulo et al., 2011).

Phages are obligate parasites that require a bacterial host for reproduction and are the most abundant and diverse biological entities on earth (Suttle, 2005). Structurally, the majority of phages are composed of a nucleic-acid genome packaged inside a protein shell (i.e., “capsid”; **Figures 1A, B**). Phage capsids are highly variable, both in size and morphology (i.e., polyhedral, filamentous, or pleomorphic). Some phages present an outer lipid membrane in addition to their protein capsid, while others only have the lipid membrane (Dion et al., 2020).

Phage genomes are variable in size (ranging between ~3.5 kb and ∼540 kb) and are composed of either single or double-stranded DNA (ssDNA, dsDNA), or RNA (ssRNA, dsRNA) (Holmfeldt et al., 2013; Dutilh et al., 2014; Norman et al., 2015; Kauffman et al., 2018; Ofir and Sorek, 2018; Sausset et al., 2020; Yang et al., 2021; Zhai et al., 2021). Consequently, the genomes of phages infecting different hosts seldom share sequence similarities (Grose and Casjens, 2014). Additionally, phage genomes present a mosaic structure as a result of recombination events with bacteria (Harrison and Brockhurst, 2017) and other phage (Dion et al., 2020). This genome mosaicism is characterized by highly similar sequences juxtaposed with sequences with which they share no apparent similarities (Hendrix et al., 1999; Pope et al., 2015; Oliveira et al., 2019). The extreme variability and mosaicism of phage genomes significantly complicate taxonomic classification, which was originally performed using shared, sequence agnostic features (e.g., capsid morphology and type of nucleic acid composing the genome) (Dion et al., 2020). For these reasons, virus taxonomy is under review due to new classification methods that include genomic sequences, genome organization, and host range (Simmonds et al., 2017). (Up-to-date viral taxonomy information can be found at https://ictv.global/vmr/). Nonetheless, the original nomenclature is still commonly used, and phages are often distinguished based on the type of nucleic acids they carry (e.g., dsDNA) and structural conformation (e.g., *Caudovirales*).

Phages are generally classified as virulent and temperate phages based on the life cycle they follow. After the recognition and subsequent attachment to a specific receptor on the bacterial cell surface, the phage delivers its genome into the bacterial cell. Here, the phage genome is replicated and expressed using host cellular resources before new complete viral particles (virions) are assembled and released from the bacterial cell. Newly assembled virions can be released by phage-mediated lysis of the bacterial cell in the lytic cycle, which is common to most known phages, or through a bacterial secretion apparatus in the chronic cycle, of the filamentous *Inoviridae* phages [**Figure 1B**; reviewed by Hobbs and Abedon (2016)]. By contrast, a temperate phage can undertake a lysogenic cycle in which, following the insertion of its genome into the bacterial cell, the phage enters a quiescent state. During this state, the phage genome, or prophage, is replicated with the host genome, either as a result of being integrated into the bacterial chromosome or as an extrachromosomal molecule. The lysogenic cycle typically ends when a specific stimulus [e.g., the bacterial SOS response (Oppenheim et al., 2005)] initiates either the lytic or chronic cycle and promotes the production of new virions and their release from the bacterial cell. However, prophages may also become defective and lose the ability to be induced or to excise from the bacterial chromosome (Wang et al., 2010; Ramisetty and Sudhakari, 2019).

### Phages in the Human Gastrointestinal Tract

The density of phages increases through the gastrointestinal tract from the small to the large intestine (Shkoporov and Hill, 2019). The density of phages in the large intestine ranges between 10^8^ and 8×10^10^ phage virions per gram of feces, measured by phage particles count (Kim et al., 2011; Hoyles et al., 2014) and estimation of viral genome numbers in feces of healthy adults (Shkoporov et al., 2019). In a mixed cohort of healthy and unhealthy individuals, phages appeared to make up the vast majority (97.7%) of gut viral genomes, with eukaryotic (2.1%), and archaeal viruses (0.1%) accounting for the remainder (Gregory et al., 2020). Notably, approximately 90% of the phage component was unclassified, while the remainder were non-enveloped DNA phages, belonging to the dsDNA order *Caudovirales* or the ssDNA families of *Microviridae* and *Inoviridae* (**Figure 1** **) (** Gregory et al., 2020).

The genomic diversity of gut phages remains largely unknown (Mirzaei and Maurice, 2017; Dion et al., 2020; Sausset et al., 2020) due to different impediments such as 1) the lack of a universal marker gene (i.e. an equivalent to the bacterial 16S rRNA gene) for targeted phage amplicon analyses and taxonomic assignments, 2) the high variability of phage genomes, 3) and the difficulty of cultivating gut phages (Rastall, 2004). The impact of these limitations is illustrated by the fact that the two most abundant fecal phage clades, crAssphage and Gubaphage, were only identified in 2014 (Dutilh et al., 2014) and 2021 (Camarillo-Guerrero et al., 2021), respectively. Both crAssphage (Cross Assembly phage) and Gubaphage (Gut Bacteroidales phage) are dsDNA phages characterized by long genomes (~ 97 and ~80 kb, respectively) which infect bacteria of the genus *Bacteroides (* Dutilh et al., 2014; Camarillo-Guerrero et al., 2021 *)*. This association has been predicted *in silico* for Gubaphage (Camarillo-Guerrero et al., 2021), but it has been demonstrated *in vitro* for crAssphage (Shkoporov et al., 2018a). *In vitro* cultivation has also revealed that crAssphage has a Podovirus-like conformation(**Figure 1B**) and a temperate life cycle (Shkoporov et al., 2018a). The discovery of crAssphage has led to the identification of numerous crAss-like phages in the human gut microbiome (Dutilh et al., 2014).

RNA phages are rare, if not absent, in the gut. Instead, the majority of viral RNA genomes within the gastrointestinal tract originate from plant viruses acquired in diet (Zhang et al., 2006; Lim et al., 2015). However, it remains possible that the low abundance of RNA gut phage genomes observed is due to the limited numbers of studies that have analysed the RNA viral component, and the scarcity of reference phage genomes for contig identification (Zhang et al., 2019; Gregory et al., 2020). Moreover, the abundance of dsDNA *Caudovirales* phages and ssDNA phages(**Figure 1**) found in the human GIT, could be due to methodological biases in virion extraction and metagenomic sequencing that favour their identification at the expense of other phages [e.g. chloroform extraction for *Caudovirales (* Thurber et al., 2009 *)* or multiple displacement amplification for ssDNA phages (Roux et al., 2016)].

The phage composition of the gut has been reported to remain stable for up to 1 year period in healthy adults (Shkoporov et al., 2019), and alterations have been associated with gastrointestinal diseases, such as *Clostridioides* (formerly *Clostridium*) *difficile* infections (CDI) and inflammatory bowel diseases (IBD) (Norman et al., 2015; Manrique et al., 2016; Draper et al., 2018; Zuo et al., 2018; Clooney et al., 2019; Park et al., 2019). Notably, different diseases (i.e., CDI and norovirus-associated diarrhoea, or ulcerative colitis and Crohn’s disease) were associated with specific gut phage compositions (Norman et al., 2015; Zuo et al., 2018). More specifically, compared to healthy subjects, CDI patients showed an increased abundance of *Caudovirales* phages and a reduction in their diversity, richness, and eveness, while norovirus-associated diarrhoea patients showed, alongside reduction in *Caudovirales* richness and diversity, also a reduction in their abundance (Zuo et al., 2018). Furthermore, Crohn’s disease patients presented an increased *Caudovirales* richness when compared to healthy controls, while ulcerative colitis patients did not (Norman et al., 2015), overall suggesting a connection between phage occupancy and the health state of the gut.

### Phage Interactions in the Human GIT and Population Dynamics

The alterations of the gut phage composition in association with different diseases suggest a potential ecological influence of phages on the gut ecosystem. This influence has been attributed to their ability to interact with and modulate the gut bacterial community and the host immune system (Mirzaei and Maurice, 2017), as both affect the homeostasis of the GIT (Chow et al., 2010; Shi et al., 2017).

The strongest contribution that phages exert on the shape of the gut ecosystem is arguably through the modulation of the gut bacterial community. This is dependent on the phage life cycle. During the lytic cycle, phage predation follows predator-prey-like dynamics (De Sordi et al., 2019). As such, there are strong selective pressures for bacteria to evolve resistance mechanisms against lytic phages (Labrie et al., 2010; van Houte et al., 2016; Mirzaei and Maurice, 2017; De Sordi et al., 2019), and for the phages to develop strategies to evade those mechanisms (Samson et al., 2013; Mirzaei and Maurice, 2017). This creates an arms race in which bacteria and phages are constantly evolving mechanisms to prevent and promote infection (Hampton et al., 2020), respectively.

However, interactions between phages and bacteria extend beyond predator-prey dynamics, as lysogenic phage infection has been suggested to potentially have beneficial effects on the bacterial host (Feiner et al., 2015). While a prophage remains dormant in the bacterial cell, its survival is directly linked to that of the host. Therefore, it is evolutionarily advantageous for the prophage to contribute to the host survival (Feiner et al., 2015), in what could be arguably referred to as a mutualistic interaction (Bronstein, 2015). Such mutualism is exemplified by virions carrying genes that have no direct impact on the phage life cycle but can enhance the fitness of the bacterial host, termed “morons” (Cumby et al., 2012) in a phenomenon called *lysogenic conversion.* Common morons include, for example, bacterial virulence (Wagner and Waldor, 2002) or metabolic genes (Breitbart et al., 2018; Zuppi et al., 2020). Alternatively, morons may provide resistance from other infecting virions, in the *superinfection exclusion* phenomenon (Bondy-Denomy et al., 2016). Interestingly, genes that enhance bacterial fitness have also been identified in virulent phages (i.e. phages that follow only the lytic cycle) (Rohwer et al., 2000; Lindell et al., 2004; Breitbart et al., 2018), suggesting that they may also promote limited bacterial survival or ‘cultivation’ to favour their reproduction.

In addition to modulating bacterial communities, phages influence the gut ecosystem by interacting directly with the immune cells and thereby modulating host immune activity [reviewed in (Carroll-Portillo and Lin, 2019; Sinha and Maurice, 2019; Van Belleghem et al., 2019)]. Phage particles can cross the epithelial barrier through a process known as transcytosis (Nguyen et al., 2017) and interact directly with the mammalian immune cells. T4 phage reduced production of reactive oxygen species from peripheral blood polymorphonuclear leukocytes and reduced NF-*k*B activity in mouse model (Górski et al., 2006; Miedzybrodzki et al., 2008) demonstrating that phage particles can exert a dampening effect on the mammalian immune system. By contrast, phage particles were also shown to stimulate an immune response in mice *via* recognition from Toll-like Receptor 9 (TLR9) (Gogokhia et al., 2019), suggesting an ambivalent effect of phages on the mammalian immune response. Importantly, studies investigating the potential uses of phage therapy in treating bacterial infections demonstrated that phage particles can stimulate the production of specific neutralizing antibodies that could dampen phage activity (Majewska et al., 2015; Hodyra-Stefaniak et al., 2015), showing a reciprocal influence between phages and the mammalian immune system in the GIT. Interestingly, specific phage-encoded proteins have been shown to modulate the interaction between bacteria and the immune system. The tail adhesin Gp12 was shown to bind bacterial lipopolysaccharide [or LPS, a bacterial endotoxin (Wang and Quinn, 2010)] and impede its recognition from the human immune system (Miernikiewicz et al., 2016). Additionally, the presence of Immunoglobulin-like domains on phage capsid was shown to mediate the binding to the intestinal mucin layer, impeding its colonization by bacterial communities and providing a non-host-derived immunity (Barr et al., 2013).

Phages’ contributions to gut ecosystem dynamics are further modulated by their biological interactions with other phages upon infecting the bacterial host. These interactions can either be antagonistic or cooperative (Domingo-Calap et al., 2020), affecting the efficacy of their reproduction and, therefore, the phage-mediated modulation of bacterial communities and host immune system. Phage antagonism occurs when two phages with a common host, either compete for receptor adsorption (Schmerer et al., 2014) or impede each other’s infection and life cycle through superinfection exclusion, described above. Also, cooperative interactions between phages have been observed in overcoming bacterial defense mechanisms, such as CRISPR-Cas systems (Chevallereau et al., 2020). Moreover, *Bacillus* phages were shown to present a communication mechanism used to determine which life cycle to follow (Erez et al., 2017; Bernard et al., 2021), highlighting the surprisingly social nature of some phages.

As a result of these multiple interactions, the human GIT is likely to be home to different population dynamics between phages and bacteria. Population dynamics is the study of the changes of a population in size and structure over time, and the factors behind them. These changes are described through mathematical models (**Box 1**).

Box 1 Models proposed to describe the phage-bacteria population dynamics can be divided into two groups:

**Group 1)** models that are characterized by a low variability and diversity of phage and bacterial populations [e. Arms-race Dynamic (Hampton et al., 2020) and Piggyback-the-Winner dynamic (Knowles et al., 2016)];

***The Arms-race dynamic*** (ARD) is characterized by competition for survival driven by predator-prey interactions, with the bacterial population developing counter-infection defenses, while the phage population develops methods to evade these defenses [Reviewed in (Hampton et al., 2020)]. This leads to an arms race that causes the extinction of not “up to date” phage and bacterial populations, while the competent ones flourish (Mirzaei and Maurice, 2017), resulting in few dominant phage and bacterial populations.

***The Piggyback-the-winner dynamic*** (PtW) is driven by mutualistic interactions that occur in the lysogenic life cycle. More specifically, describing prophages that contribute to the survival of hosts through lysogenic conversion and superinfection exclusion. This dynamic is characterized by low variability and diversity of the phage and bacterial populations (Knowles et al., 2016; Mirzaei and Maurice, 2017) and leads to a few populations out-competing the others thanks to the advantages provided by this mutually beneficial interaction. PtW is the only proposed model of phage-bacteria population dynamics that is connected to the lysogenic cycle.

**Group 2)** models that are characterized by high phage and bacterial population variability and diversity [i.e. Fluctuating selection dynamic (Hall et al., 2011) and Kill-the-winner dynamic (Thingstad, 2000)]. These models are driven by “negative frequency-dependent selection”, a form of natural selection in which the fitness of a genotype is inversely proportional to its frequency (Clarke, 1962).

***The Fluctuating-selection dynamic*** (FSD) results from the bacterial fitness costs for developing phage-resistance mechanisms being disadvantageous in an environment in which multiple bacterial species are competing for resources. Specifically, effective phage-resistance mechanisms lead to a decrease in the number of infecting virions. As predation decreases, phage-resistant bacterial populations will be outcompeted by bacterial populations which did not invest in defense mechanisms. Ultimately, this leads to a switch in the bacterial communities from phage-resistant to non-phage-resistant bacteria. In this newly permissive environment, phage predation increases, making the niche favorable again for the growth of phage-resistant bacteria populations, and the cycle begins anew (Avrani et al., 2012).

***The Kill-the-winner dynamic*** (KtW) occurs when the abundance of the “winning” bacterial species (i.e., the most competitive) is controlled by phage predation. This allows the coexistence of multiple bacterial and phage populations by limiting the expansion of the most competitive populations (Thingstad, 2000).

Alternatively, these population dynamics models can be grouped based on the prevalent phage life cycle and modalities of interaction between phages and bacteria populations in the environment, namely lytic or predator-prey interactions (ARD, KtW, and FSD) and lysogenic or mutualistic interactions (PtW). The type of life cycle followed by phages in an environment is inferred based on the Virus-to-Microbe ratio (VMR), as VMR lower than one suggests the prevalence of the lysogenic cycle, while higher VMR suggests the prevalence of the lytic cycle.

Population dynamics are strictly dependent on the environment in which they take place. In GIT, factors influencing the population dynamics include pH and bile acid levels, structural conformations, mucin layer, oxygen levels, and nutrient availability; all varying across the length of the GIT. This variability influences gut bacteria (Donaldson et al., 2016) and, in certain cases, phage populations (Verthé et al., 2004; Ma et al., 2008; Jończyk et al., 2011). Mirroring these physiological gradients, phage-bacterial population dynamics have also been reported to vary throughout the gut (Shkoporov and Hill, 2019). For example, the prevalent population dynamic in the lumen of the colon is thought to be the PtW dynamic, as suggested by the low VMR reported in feces (≥ 1:1) (Reyes et al., 2010; Shkoporov et al., 2018b). In the mucin layer of the colon, the high VMR (~20:1) (Barr et al., 2013) suggests lytic cycle-driven dynamics (e.g. ARD, KtW, or FSD) (**Box 1**). This has been connected to a reduction of bacterial cell densities in the mucin layer compared to the lumen of the colon (Shkoporov and Hill, 2019). This is consistent with what was reported by Knowles et al., who showed that, in marine environments, the prevalent life cycle in a phage population was connected to bacterial densities (Knowles et al., 2016). Similarly, it was suggested that the prevalent population dynamic changes toward the distal part of the colon, as an increasing number of stressors reduce bacterial densities and promote induction of prophages, resulting in a shift toward lytic cycle-driven dynamics (ARD, KtW, or FSD) (Shkoporov and Hill, 2019).

Importantly, the population dynamics in the human GIT are likely to differ from the models used to describe them, as these were originally proposed for other environments [i.e. *in vitro (* Hall et al., 2011 *)* and aquatic environments (Thingstad, 2000; Knowles et al., 2016)], which are significantly different from the human GIT (Shkoporov and Hill, 2019). This has been exemplified by Park et al. who reported that, in patients with recurrent CDI, an increase in gut phageome (i.e., the phage component of the microbiome) diversity was accompanied by a reduction of bacterial diversity, contrary to predictions from the proposed models (**Box 1**) (Park et al., 2019). Despite being unclear whether these changes in microbial diversity might be the cause or the results of CDI, the increase of phage diversity not being coupled by an increase in bacterial diversity suggests that other factors in the human GIT contribute to these dynamics. These modifiers of the phage-bacteria population dynamics may include the host immune system, as it has been shown to both strongly influence and be influenced by both phages and bacteria. This hypothesis is supported by observations from Clooney and colleagues who identified an increase in induced temperate phages in IBD patients and connected the phage induction to an increase in inflammation biomarkers, such as reactive oxygen species (Clooney et al., 2019).

### The Ecological Role of Phages in the Gut Ecosystem

**Figure 2:**
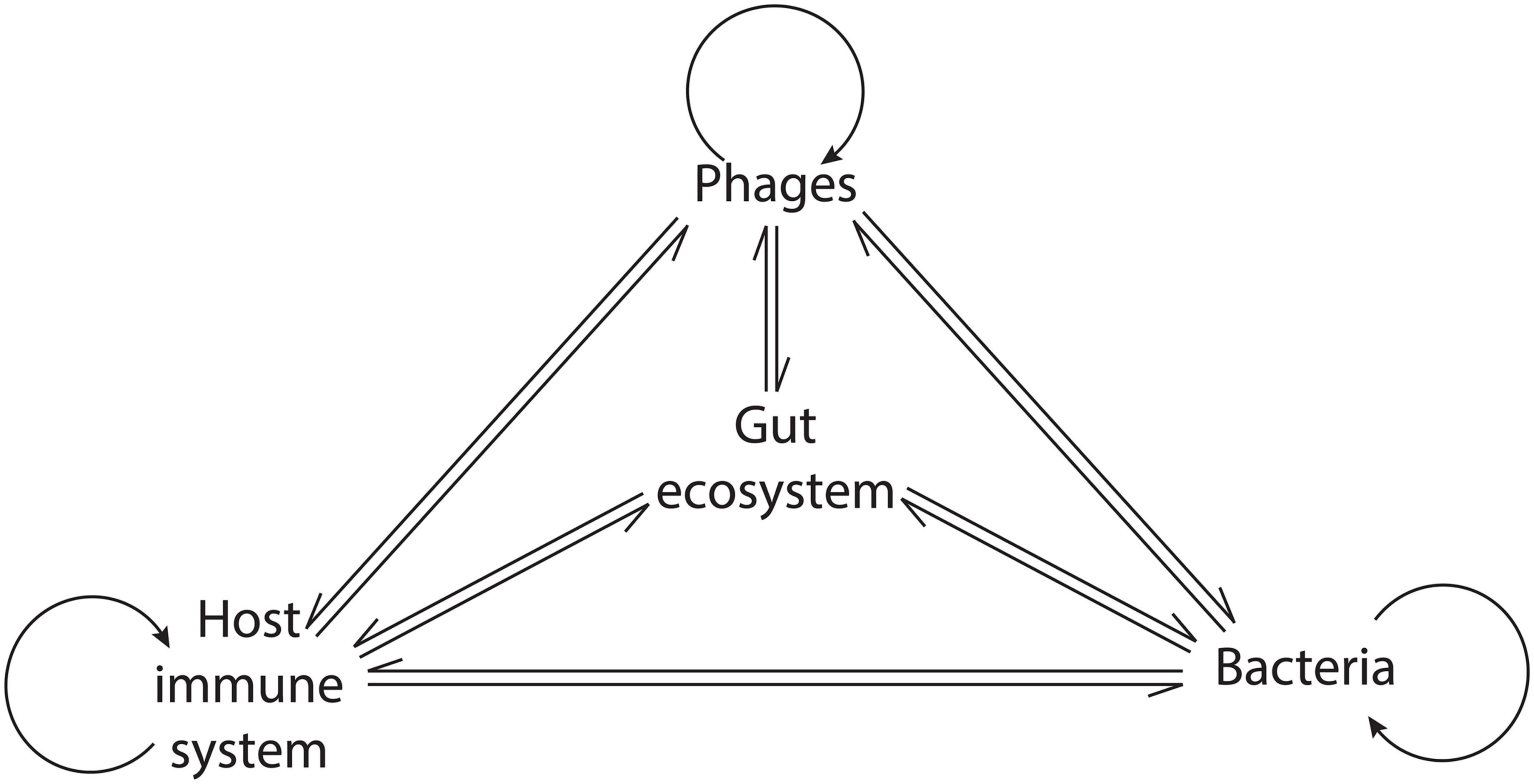
Network of the phage interactions in the gut ecosystem. Phages exert a direct influence on bacteria, the host immune system, and themselves. Indirectly, their activity modulates the interaction within bacterial communities and between bacteria and the host immune system, leading to cascading ecosystem effects.

The organisms in macroscopic ecosystems are connected by physical interactions and by participation in the flux of materials and energy (Stuart Chapin et al., 2011). Similar to macroscopic ecosystems, Hsu and colleagues illustrated the connection between different organisms in the gut by showing that, in gnotobiotic mice, phage-mediated lysis of bacterial populations had cascading effects on bacterial populations that were not directly targeted by phage predation (Hsu et al., 2019). Fluctuations in the abundance of individual species have distinct consequences on the ecosystem depending on their ecological role (Stuart Chapin et al., 2011). In the human GIT, as resident microbial communities exert a strong influence on the host immune system, phage-mediated alterations of these communities could promote cascading effects potentially leading to profound ecosystem alterations and disease (Tetz and Tetz, 2018) (**Figure 2**).

Phages alter the gut commensal bacterial composition through their parasitic activity. This may lead to the development of gastrointestinal diseases through altered colonization resistance and proliferation of opportunistic or obligate pathogens in vacated niches (Stecher and Hardt, 2008). Furthermore, phage-mediated depletion of commensal bacterial populations may interfere with the production of immunomodulatory compounds. IBD patients were shown to have reduced levels of *Faecalibacterium prausnitzii (* Sokol et al., 2009 *)* and increased levels of its phage (Cornuault et al., 2018). Notably, *F. prausnitzii* is a major contributor of colonic butyrate (Segain et al., 2000), which is an anti-inflammatory short-chain fatty acid (SCFA) (Liu et al., 2018). Therefore, increased phage predation may indirectly contribute to IBD inflammation by reducing the amounts of butyrate produced by *F. prausnitzii*.

Similarly, Parkinson’s disease patients were observed to have increased levels of lytic *Lactococcus* phages and depleted levels of *Lactococcus* bacteria, the latter of which regulate gut permeability and produce dopamine. These factors are implicated in Parkinson’s disease pathogenesis, suggesting that phage predation within the GIT could contribute to the development of this disease (Tetz et al., 2018). More broadly, these data demonstrate that phage-mediated depletion of commensal bacterial populations may lead to decreased production of bacterially-derived immunomodulatory compounds, such as SCFAs, thereby modulating gut inflammation. By contrast, phage predatory activity may contribute to GIT health by keeping bacterial abundances within tolerable levels. Consistent with this, phages were shown to provide a non-host derived immunity by binding to the mucin layer surrounding the intestinal epithelium *via* an Ig-like protein in the capsid. Through their lytic activity and predation in the mucin layer, phages prevent its colonization from bacterial cells (Barr et al., 2013) and activation of the immune system (Wells et al., 2017).

Temperate phages are capable of strongly contributing to the bacterial host’s virulence and fitness by providing virulence genes, such as the phage-encoded toxins or immune evasion genes (Penadés et al., 2015). In this way, temperate phages can indirectly exert a pro-inflammatory effect in the GIT and cause alterations to the gut ecosystem. Prophages may also increase the fitness of commensal bacteria through superinfection exclusion or lysogenic conversion, contributing to the maintenance of a healthy gut environment. Importantly, the ecological impact of temperate phages goes beyond increasing the fitness of the bacterial cells they infect. Prophages can be induced, leading to the initiation of the lytic cycle and the death of their bacterial host, potentially changing the population dynamics and the impact on the GIT. Therefore, stimuli that cause prophage induction can affect their influence on the gut ecosystem. Following this reasoning, the induction of prophages has been proposed to participate in the establishment of positive feedback loops of GIT inflammation in humans (Lin and Lin, 2019).

**Figure 3:**
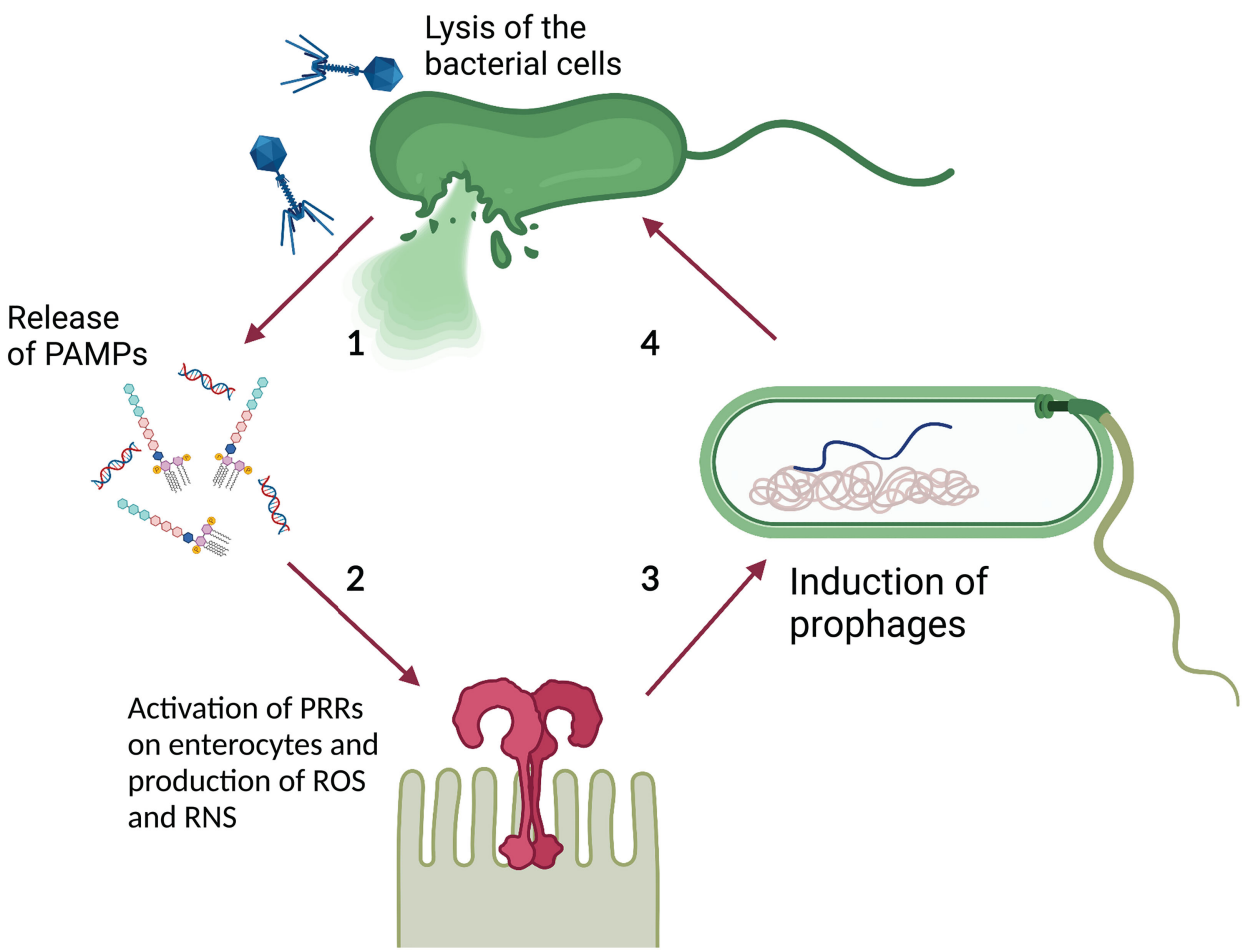
Theoretical model of phage-PAMP-PRR mediated positive feedback loop for GIT inflammation. **1)** Phage-mediated bacterial lysis causes the release of PAMPs, such as bacterial DNA and LPS. **2)** PAMPs are recognized by PRRs, such as TLR, located on the surface of intestinal epithelial cells, leading to the production of ROS and RNS. **3)** ROS and RNS stimulate prophage induction directly, by damaging bacterial DNA and activating the SOS response, or indirectly, by stimulating the inflammatory response. The inflammatory response increases the luminal oxidative stress causing damage to the DNA and activates the SOS response. **4)** The induction of the dormant prophage causes the initiation of the phage lytic cycle resulting in the lysis of the bacterial cell. Image created with Biorender.com.

Lin et al. theorized that prophage induction promotes gut inflammation by spreading integrated virulence factors. Gut inflammation augments intestinal permeability, increasing the luminal oxygen level. This in turn promotes prophage induction through a mechanism that involves oxidative stress establishing a positive feedback loop (Lin and Lin, 2019). Similarly, Clooney et al. (2019) suggested that phage-mediated lysis of bacterial cells contributes to IBD associated gut inflammation by promoting the release of Pathogen Associated Molecular Patterns (PAMP), such as bacterial DNA, lipopolysaccharide, and peptidoglycan (Tetz and Tetz, 2018), and their exposure to Pattern Recognition Receptors (PRRs) on gastrointestinal epithelial cells (e.g. Toll-like Receptors or TLR) (Takeuchi and Akira, 2010). This triggers the production of Reactive Oxygen and Reactive Nitrogen Species (ROS and RNS, respectively) which promote a host inflammatory response (Rokutan et al., 2008) that can indirectly increase prophage induction, as suggested previously. Moreover, ROS and RNS can promote prophage induction directly (Łoś et al., 2010; Diard et al., 2017), suggesting yet another mechanism through which phages may promote a positive inflammatory feedback loop (**Figure 3**).

The intense predatory activity of phages can have profound influences on the microbial communities and the gut ecosystem at large. Therefore, phages have been compared to apex predators in macroscopic environments (Hsu et al., 2019), namely predators whose activity significantly shapes the predated ecosystem. Nonetheless, the ambivalence of the interactions of temperate phages with bacterial communities, which can occur both in a predator-prey and mutualistic manner, suggests that their ecological role is more complex. Intriguingly, the modality of interaction with bacterial communities, and therefore the interaction with the host immune system and the population dynamics, appear to shift in response to environmental changes. This suggests that the ecological role of phages changes depending on the surrounding environment.

The known complexity of the direct and indirect impacts of phages on the gut ecosystem suggests that their ecological role has no direct comparison in the macroscopic environments. However, it is clear that phages strongly contribute to the temporal patterns, directionality, frequency, and magnitude of population changes within the gut microbial community, with profound repercussions on the gut ecosystem and health.

## Future Research

**Figure 4:**
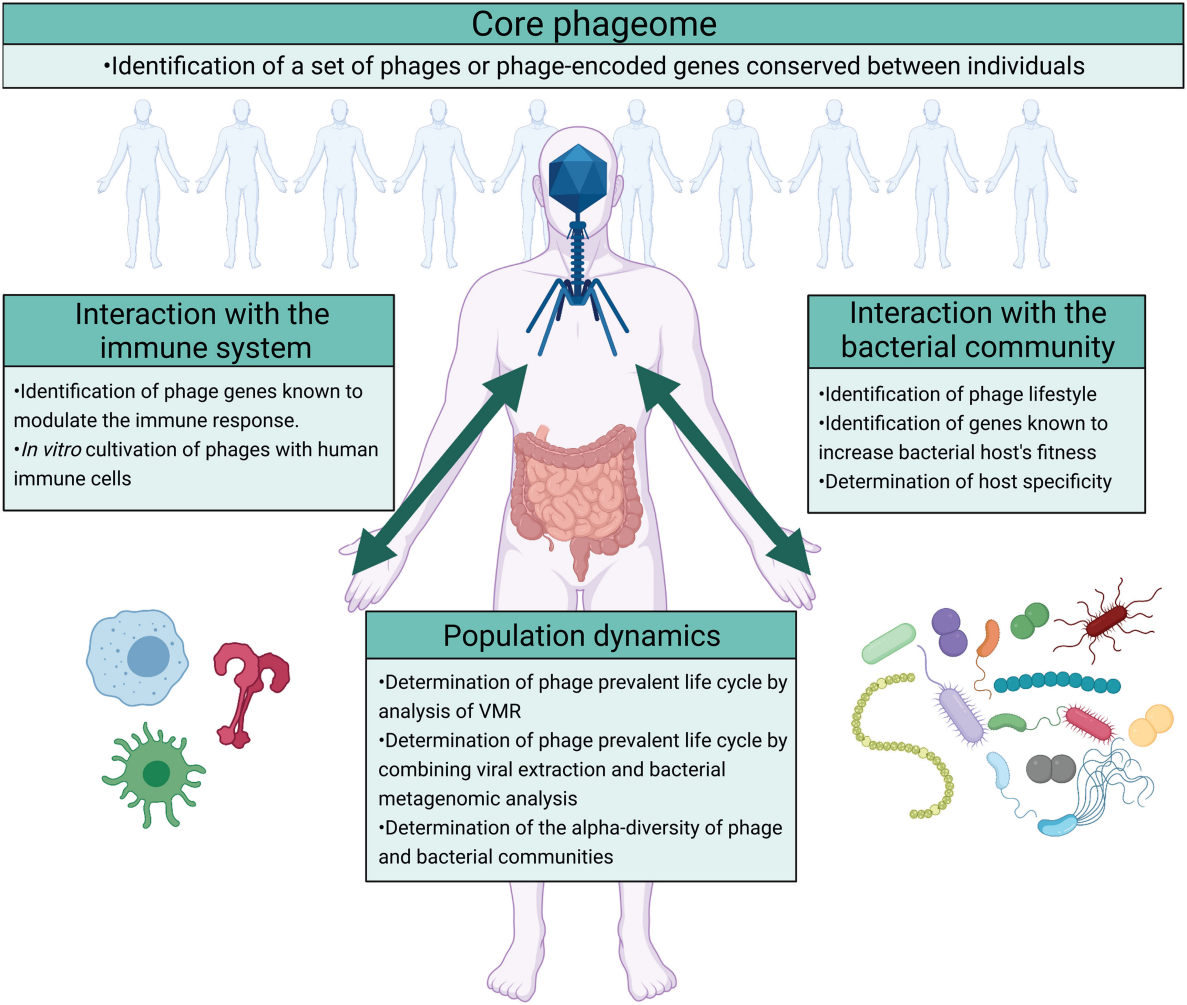
Future directions on the study of the ecological roles of phages in the gut ecosystem. The impact of phages on the gut ecosystem can be studied by focusing on four different questions regarding the existence of a core phageome, phage’s interaction with the bacterial community, phage’s interaction with the host immune system, and the population dynamics that result. The identification of a core phageome would simplify the study of the gut phageome allowing a more targeted analysis. Moreover, the impact of phages on the gut ecosystem can arguably be identified by analyzing their interaction with the bacterial community and the host immune system. In addition to determining the interaction between phages, the bacterial community, and the immune system, studying the population dynamics that results would describe the outcomes of these interactions. In the figure are suggested different methods that can be used to answer these questions. Image created with Biorender.com.

​​Despite the increasing interest in gut phages and their roles in the gut microbiome, research into this field is still in its infancy. Arguably, a key question is the role of the phageome in the gut microbial ecosystem at large. This can be further divided into more specific questions regarding 1) consistency or variability of gut phageomes across individuals and populations; 2) host-specificity and influence of gut phages on their bacterial host; 3) direct influence of gut phageome on the human immune system; 4) shifts in the gut population dynamics either driven by or contributed by individual phage or phages(**Figure 4**).

To begin addressing these questions, it is important to survey the diversity and stability of gut phage composition across populations (rural, urban and international; healthy and unwell). This would allow the determination of a “core phageome” (i.e., a set of phages consistently identified in the gut microbiomes of individuals) and those that are positively associated with gut health. Identifying such a set of phages would allow a more targeted analysis, facilitating the understanding of the role of phages in the gut microbiome. Current attempts to address this question have reported highly subject-specific phageomes with only a minimal proportion of phage genomes (less than 1%) shared across more than half of the studied population (Manrique et al., 2016; Gregory et al., 2020). As phage genomes are highly mosaic and variable, a “core phageome” could be better identified by focusing on shared phage functions between individuals, rather than attempting to identify common phage taxa or genomes. While most phageome genes are involved in the phage life cycle or encode structural proteins (Nayfach et al., 2021), morons are involved in bacterial phenotypes and ecosystem dynamics in a variety of ways. Targeted identification and annotation of morons could therefore provide an additional view to how conserved or individualized the phageomes are across individuals and populations (i.e., core functional phageome). Since homology-based methods can only annotate a minority of phage gene clusters (Nayfach et al., 2021), functional characterization of phageomes will benefit from recent neural network-based protein structure and function prediction methodologies (Baek et al., 2021; Gligorijevic et al., 2021; Jumper et al., 2021; Tunyasuvunakool et al., 2021). Specific morons to evaluate are genes that increase the fitness of bacterial hosts. These include, but are not limited to, antibiotic resistance genes [from databases such as CARD (Alcock et al., 2020) or ARDB (Liu and Pop, 2009)], virulence genes [VFDB (Chen et al., 2005), MvirDB (Zhou et al., 2007)], genes implicated in bacterial motility and other auxiliary metabolic genes (AMGs); specific tools to assess metagenomic AMGs include DRAM-v (Shaffer et al., 2020) and VIBRANT (Kieft et al., 2020). Additionally, other databases and tools developed for cataloging or analyzing prokaryotic genomes, such as DRAM (prokaryotic version) and METABOLIC (Zhou et al., 2020), could enable the identification of additional morons that were overlooked by the phage-specific tools or databases above. Genes coding for structural proteins can also be included in the set of phage-encoded genes that impact the gut ecosystem. An example of this is provided by the tail protein Gp12 (Miernikiewicz et al., 2016) or capsid Ig-like domain (Barr et al., 2013), structural proteins with potential anti-inflammatory outcomes.

Identifying such genes in the phageome also allows the determination of the impact that phages have on the gut ecosystem. The presence of lysogenic conversion morons in a phage genome describes its potential influence on its bacterial hosts, while the presence of genes such as the ones coding for tail protein Gp12 or the capsid Ig-like domains describe its potential influence on the human immune system. Nonetheless, the role of phages in the gut ecosystem is not entirely dependent on the morons or the immune-modulation genes they encode, but it is also dependent on the different life cycles they can follow and the bacterial hosts they infect. The influence that phages have on the bacterial host is in fact strictly dependent on their lifestyle, as temperate phages can establish a mutualistic relationship with their host, while virulent phages establish a predator-prey relationship. In addition to this, the ecosystemic outcomes of the phage influence on the bacterial host depend on the ecological role of that host. For example, a temperate phage increasing the fitness of the bacterial host by carrying AMGs, or a virulent phage infecting a bacterial population, will impact the gut ecosystem differently depending on whether the bacterial host is a commensal or a pathogenic bacterium. Moreover, phages able to infect multiple hosts can favour the spread of virulence genes with negative outcomes on gastrointestinal health. To investigate the lifestyle and host-specificity of phages, multiple tools have been developed. To determine the lifestyle of the sequenced phages, tools such as BACPHLIP (Hockenberry and Wilke, 2021), Deephage (Wu et al., 2020), or PHACTS (McNair et al., 2012) can be used. BACPHLIP determines the phage lifestyle based on the presence or absence of genes associated with the lysogenic cycle in its genome. Deephage and PHACTS predict the phages’ lifestyle by identifying, in their genome, nucleotide features shared with a set of phages with known life cycle. To determine the host specificity of the sequenced phages, different tools have been proposed, and have been collectively described in ref (Coclet and Roux, 2021). These approaches can be divided into 1) alignment-dependent approaches, which determine the host specificity of a phage by identifying the sequences it shares with either bacteria [e.g., SpacePHARER (Zhang et al., 2021)] or phages with known hosts [e.g., RaFAH (Coutinho et al., 2021)] in databases; 2) alignment-free approaches, which determine the host specificity based on genomic convergence between a phage and its host through machine learning models [e.g., WIsH (Galiez et al., 2017) or PHP (Lu et al., 2021)]; 3) integrative approaches that combine alignment-dependent and alignment-free approaches [e.g., VirHostMatcher Net (Wang et al., 2020) or PHISDetector (Zhang F et al., 2020)].

These approaches present significant impediments, as determining the phage lifestyle based on the presence or absence of specific genes is strictly dependent on the completeness of the assembled contig. Differently, tools that, to assess the phage lifestyle, rely on nucleotide similarities between the query and a set of phage genomes might exclude phages that significantly differ from that set. Furthermore, the tools to determine the host specificity present impediments based on the prediction approach. Alignment-based approaches strongly depend on the reference databases, while alignment-free approaches have a high occurrence of false positives, and the results often need to be confirmed through statistical analysis. Integrative approaches appear to overcome these issues but are still relatively new and their reliability still needs to be assessed (Coclet and Roux, 2021). Moreover, none of the different approaches that predict the host specificity describe the efficacy of the infection, i.e., the duration of the lytic cycle and the number of virions produced. The efficacy of phage infection has been reported to vary depending on the bacterial host infected (Breitbart et al., 2018), with potentially different outcomes on the ecosystem. While numerous tools are being developed to determine the phage host specificity, a similar effort is not being made in the prediction of the phage lifestyle. The currently available tools either present significant impediments (i.e., BACPHLIP requires the query contigs to represent complete genomes) or are not user-friendly (i.e., Deephage). PHACTS is a web-based tool, which is not suitable for metagenomic analysis of large data. Therefore, the development of bioinformatic tools to predict the phage lifestyle that are more apt at metagenomic analysis would strongly contribute to optimizing the study of gut phageome.

Alternatively, in addition to *in silico* analyses of metagenomes, targeted *in vitro* work could contribute to the validation of computationally derived predictions of phage-host interactions, phage lifestyle, phage host-specificity, and efficacy of phage infection. A significant impediment to *in vitro* cultivation of gut phages is posed by the lifestyle of most gut bacteria, which are facultative or obligate anaerobes (Sherwood et al., 2013). Nonetheless, different approaches for *in vitro* cultivation have been provided to overcome this issue (Shkoporov et al., 2018a; Forster et al., 2019; Sardelli et al., 2021). Moreover, *in vitro* and *in vivo* description of phage interactions with human immune cells holds promising potential to study their impact on the host immune system in the GIT, as suggested by previous results (Miedzybrodzki et al., 2008; Miernikiewicz et al., 2016; Gogokhia et al., 2019).

Changes in phageomes and phage-induced changes in bacteria may, in extreme cases, reorganize the gut ecosystem through shifts in population dynamics. Population dynamics in an environment have been usually described by determining the alpha-diversity of phage and bacterial populations and the VMR, an indication of the prevalent phage life cycle. However, analyzing gut ecosystems using sequencing-based technologies overlooks both changes in absolute virion and bacterial cell abundances as well as different microenvironments within the gut. To overcome the former limitation, measuring absolute viral abundance using a separate assay, such as quantitative microscopy (Liang et al., 2020) or phage spike-ins (Shkoporov et al., 2018b), would help assess changes in population dynamics. To further address this issue, researchers should consider conducting, alongside purification and metagenomic sequencing of phages, also metagenomic sequencing of the bacterial component of the same sample. This can be performed either through direct analysis of the isolated bacterial component following viral enrichment. Such analysis will capture and distinguish between phages undergoing the lytic cycle and dormant prophages integrated into bacterial genomes. Together with assessing the alpha diversity of phage and bacterial communities in a gut microbiome, identifying the prevalent life cycle would help identify the population dynamics between the two (Knowles et al., 2016). Finally, integration of phageome and bacteriome analysis also enables the construction of bacterial CRISPR arrays (bacterial immune memory against phages) which can be compared to the phage genome repertoire. Evaluating the composition and evolution of bacterial CRISPR spacers could provide additional phage-host pair prediction. Together with other tools specifically designed for evaluating bacterial anti-phage defense systems, such as PADS Arsenal (Zhang Y et al., 2020), such analyses will provide insights into the phage-bacteria arms race, other interactions, and gut ecosystem dynamics at large.

Overall, improving computational phageome analyses, integration of phage virions and bacterial metagenomes with *in vitro* validations and follow-up studies will lead to a comprehensive understanding of the gut phageome, ultimately revolutionizing the way we think about our inner ecosystems.

## Conclusions

Phages are pivotal components of the human gastrointestinal tract and changes in the phage composition and abundance have been associated with multiple gastrointestinal diseases. They are major drivers of bacterial evolution and important modulators of the host immune system, thereby contributing to the gut ecosystem dynamics. Despite much is yet to be understood about the gut phageome, focusing on the interactions between phages, gut bacteria and the host immune system holds promising potential for a more complete understanding of the gut microbiome and its connection with human health.

## Author Contributions

MZ wrote the manuscript with comments from all other authors. JO’S and TV supervised the work. All authors contributed to the article and approved the submitted version.

## Funding

This work was funded by The Royal Society Te Apārangi Marsden Fund (MFP-UOA1901).

## Conflict of Interest

The authors declare that the research was conducted in the absence of any commercial or financial relationships that could be construed as a potential conflict of interest.

## Publisher’s Note

All claims expressed in this article are solely those of the authors and do not necessarily represent those of their affiliated organizations, or those of the publisher, the editors and the reviewers. Any product that may be evaluated in this article, or claim that may be made by its manufacturer, is not guaranteed or endorsed by the publisher.
